# A Selection of 14 Tetrameric Microsatellite Markers for Genetic Investigations in Fallow Deer (*Dama dama*)

**DOI:** 10.3390/ani13132083

**Published:** 2023-06-23

**Authors:** Orsolya Krisztina Zorkóczy, Orsolya Turi, Zsombor Wagenhoffer, László Ózsvári, Pál Lehotzky, Zsolt Pádár, Petra Zenke

**Affiliations:** 1 Nutrition and Laboratory Animal Science, Department of Animal Breeding, University of Veterinary Medicine Budapest, H-1078 Budapest, Hungary; torsi2001@gmail.com (O.T.); wagenhoffer.zsombor@univet.hu (Z.W.); 2 Department of Veterinary Forensics and Economics, University of Veterinary Medicine Budapest, H-1078 Budapest, Hungary; ozsvari.laszlo@univet.hu; 3 National Laboratory of Infectious Animal Diseases, Antimicrobial Resistance, Veterinary Public Health and Food Chain Safety, University of Veterinary Medicine Budapest, H-1078 Budapest, Hungary; 4 Hungarian Hunters’ National Chamber, H-1027 Budapest, Hungary; 5 Department of Criminal Sciences, Ferenc Deák Faculty of Law and Political Sciences, University of Győr, H-9026 Győr, Hungary

**Keywords:** fallow deer (*Dama dama*), wildlife and population genetics, tetrameric microsatellites (tetra-STRs), individual identification

## Abstract

**Simple Summary:**

Monitoring and maintaining genetic diversity is essential for the conservation of most species, including fallow deer. Various markers can be tested to estimate genetic diversity. For this purpose, microsatellites consisting of four-base-pair repetitive units are highly recommended due to their polymorphisms and comparable and reliable detection during analyses. In this study, nearly one hundred tetrameric microsatellites were collected from nine other deer species, which were tested on twenty individuals from five Hungarian fallow deer populations. As a result, 14 polymorphic markers were selected for the investigation panel, which fills a gap in the field of experts dealing with fallow deer genetics.

**Abstract:**

The fallow deer (*Dama dama*) represents significant game management value globally, and human activities are significantly impacting the species. Besides the positive effects, these activities can threaten its existence, health, and value. The aim of the authors was to develop a tetranucleotide microsatellite panel that could be clearly interpreted and used for genetic testing of fallow deer. Such a panel did not exist until now and could be particularly useful in the field of conservation genetics and forensics. A total of 99 tetrameric microsatellites, originally designed for related deer species, were tested on 20 fallow deer individuals from five Hungarian sampling areas. Original and newly designed primers were used to amplify the microsatellite regions using previously published or optimized PCR protocols. The lengths and sequences of specific amplicons were detected using capillary electrophoresis, and the rate of polymorphism was determined. Altogether, 80 markers provided PCR products of adequate quality and quantity. Among them, 15 markers proved to be polymorphic (2–5 alleles/locus), and 14 tetrameric markers were selected for further analysis. Statistical calculations showed that the selected polymorphic microsatellites can potentially enable key individualization in many areas of wildlife and population genetics, thus protecting the species.

## 1. Introduction

The fallow deer (*Dama dama*) is one of the most widely spread deer species in the world and is commonly found as an introduced mammal in Europe [1,2,3]. It holds great value not only in wildlife management due to venison, antler trophies, and the infrastructure built around deer hunting, but also in terms of cultural and conservation importance. After being (re)introduced to most European countries and other parts of the world, the increase in its population has triggered a rise in hunting efforts. In fact, the fallow deer population in Europe has increased five-fold from 1984 to the early 2020s, leading to a six-fold increase in harvest during the same period [4]. This correlation also predicts an increasing trend in possible poaching, traffic accidents, and car damages caused by deer–vehicle collisions, in which the fallow deer is frequently involved [5,6,7,8].

The monitoring and maintenance of genetic diversity is essential for the conservation of the species [9,10], especially in habitats that are more affected by humans [11]. As low levels of variance and genetic homogeneity within local populations may promote genetic degradation, fertility reduction, and the spread of infectious diseases in populations [12,13], genetic studies of fallow deer are carried out worldwide to decipher the population’s structure and health [14,15,16]. To estimate genetic diversity in this species, different markers have been tested, such as mitochondrial DNA [14,17], blood and tissue proteins [16,18,19], various genes (e.g., PRNP) [15], as well as di- and tri-nucleotide microsatellites [14,20]. Microsatellite marker-based developments have been predominantly carried out in other deer species to assess genetic diversity [21,22,23,24,25,26,27,28,29,30], as the number of repeats in microsatellites tends to be highly variable within a population. Microsatellites or short tandem repeats (STRs) are polymorphic fragments of DNA containing a repeated sequence of generally 2–5 nucleotides. Tetrameric (tetra-nucleotide) markers, with four-base-pair repeats, are strongly recommended due to their frequent occurrence and reliable detection during analyses [21,22,23,24,25,28,29,31]. Di- and tri-nucleotide microsatellites, which are shorter than tetrameric markers, can be less clearly defined, as they are more likely to result in an artifact during the polymerase chain reaction (PCR) [21,25,31]. STRs consisting of units longer than tetrameric are not so common in the genomes of more complex organisms [21] and typically exhibit low allelic variation. Additionally, population genetic parameters can also be influenced by differences in the number of markers and marker sets used, which reduces comparability between scientific data [32].

The first step in compiling a microsatellite set for genetic analysis is the search for suitable markers. This can be done by examining the genome sequence of the target species using next-generation sequencing technology [28,29,30] or by testing markers described in previous publications for closely related species [22,23,25,33]. The latter method, traditionally used due to its lower cost, may encounter the problem of primers originally designed for different species not functioning in the target species. However, studies that have dealt with multiple deer species have also found the functionality of primers in more distantly related species [25,29,33,34].

Capillary electrophoresis is the most commonly used technique for detecting polymorphic alleles of microsatellites. This technique requires fluorescent labeling of PCR products during amplification [35]. The traditional method involves directly labeling the 5′-end of the forward primers with a fluorophore dye [36,37]. When testing a large number of potential markers and their primers, a cost-saving method known as the end-labeling technique can be used, which utilizes three primers per marker [38]. In this method, in addition to the conventional reverse primer, a forward primer with a 15–18 base-long adapter sequence and another sequence (the same as the adapter) carrying a fluorophore (known as the universal primer) are added to the reaction [38]. This approach allows multiple primers/markers to be tagged with the fluorescently labeled adapter in a cost-effective, reversible, and interchangeable manner. As a result, primers/markers can be added and combined as needed during the tests (with the appropriate adapter sequence).

Tetramer-STR marker sets, which offer numerous advantages over dimeric microsatellites [21,22,23,29,30,39], have already been developed for various deer species but are not yet available for fallow deer. The aim of this study was to develop an investigative method to test all available cross-species tetrameric repeat STRs on fallow deer samples and select polymorphic markers to expand the testing options for this globally important game species.

## 2. Materials and Methods

### 2.1. Marker Selection and Primer Design

As a genome sequence is not yet available for fallow deer, a total of 107 cross-specific STR markers with tetrameric units in the family *Cervidae* were chosen from previous publications and the NCBI GenBank database (https://www.ncbi.nlm.nih.gov/genbank/, accessed on 14 February 2021) for preliminary in silico marker selection (Figure S1). Firstly, the sequences of the markers available in the NCBI GenBank database were examined for possible matches using BLAST^®^ (https://www.blast.ncbi.nlm.nih.gov/Blast.cgi, accessed on 14 February 2021) to avoid redundant testing of the same locus published under different names in separate articles (Table S2). In cases of duplicates, if multiple primer sequences were available, the loci were tested with all described primer pairs, and the optimal working pair was selected for further investigation. Three aspects were taken into consideration: the absence of by-product formation, the generation of an adequate amount of product, and the production of an amplicon with the shortest possible length (due to the often highly degraded DNA samples, such as those found in forensic investigations).

Primers were designed using the Primer Designer 4 software (http://www.scied.com, accessed on 14 February 2023) for 21 markers that did not have published primer sequences available. The design was based on deer sequences obtained from the NCBI GenBank database (Table S3). During the primer design process, factors such as amplicon size, GC content, absence of self-complementarity (hairpins), and absence of complementarity to the other primer pair (more than four base pairs at the 3′ end) were taken into consideration. All 99 forward primers (75 original and 21 designed) used for testing were synthesized and ordered with one of the four universal adapters [40]. These adapters allowed for the use of fluorescent end-labeling in subsequent analyses (Table S4). When selecting the appropriate adapters, the possibility of later parallel detection of several markers was considered.

### 2.2. Sample Collection and DNA Extraction

Muscle or hide samples (12 females and 8 males) were collected from registered shootings by hunters with a license between 2019 and 2022 from five regions in Hungary (Figure S1). Genomic DNA was isolated using a FavorPrep^TM^ Tissue Genomic DNA Extraction Mini Kit (Favorgen Biotech, Ping-Tung, Taiwan) following the provided procedural guidelines. The quality of the extracted DNA was tested using a 1% agarose gel stained with GelRed^TM^ Nucleic Acid Gel Stain (Biotium, Fremont, CA, USA), and the concentration was measured using a Qubit 2.0 Fluorometer (Life Technologies Corporation, Carlsbad, CA, USA). Isolated DNA from the tissue samples were stored at −20 °C until subsequent analysis.

### 2.3. Singleplex PCR Amplification, Optimization, and Primer Redesign

Altogether, 99 markers were selected and amplified on two fallow deer samples in singleplex reactions to determine functionality. Forward primers with universal adapters were fluorescently labeled using the end-labeling technique with four different colored fluorophores (Table S5) [38,41]. The PCR reactions (10 μL in volume) consisted of 2 μL DreamTaq™ Green PCR Master Mix (ThermoFisher Scientific, Waltham, MA, USA), 0.1 μL of BSA (20 mg/mL, Sigma–Aldrich, St. Louis, MO, USA), 0.5 μM forward and 0.5 μM unlabeled reverse primer, 1 ng DNA template, and PCR grade-H2O to volume. The PCR programs used were those published for the original source species and were carried out in an Applied Biosystems 2720 Thermal Cycler. Qualitative assessments of singleplex amplifications were conducted using 2% agarose gel stained with GelRed^TM^ Nucleic Acid Gel Stain (Biotium, Fremont, CA, USA).

In cases where the PCR settings resulted in inadequate amplicons, such as the formation of by-products, significant deviations from the expected size, and poor quantity or absence of product, the PCR settings were modified and optimized using a Mastercycler nexus GX2 (Eppendorf AG, Hamburg, Germany) gradient thermal cycler (Table S5). All PCR products resulting from the modified settings were visualized on 2% agarose gel.

If the modification of the PCR settings did not yield satisfactory results, the primers were redesigned using Primer Designer 4 software, taking into consideration the aspects mentioned in Section 2.2. The redesigned primers were then tested through singleplex PCR amplification, following a similar procedure as described earlier in this chapter (Table S6).

### 2.4. Estimation of the Polymorphism Level of the Functional Markers

To further analyze the markers, a total of 80 markers that produced suitable amplicons were tested to estimate their level of polymorphism. The functional microsatellite markers were amplified in singleplex reactions using 20 fallow deer samples, following the procedure outlined in Section 2.3 (Tables S4–S6).

#### 2.4.1. Capillary Electrophoresis

Parallel detection of 4–8 PCR products with different sizes or different fluorescent labels was possible through capillary electrophoresis. The mixing ratio of the amplicons was determined based on the intensity of their bands on the semiquantitative agarose gel (Table S7). The amplified fragment mixes were analyzed and sorted using an ABI Prism 3500XL Genetic Analyzer using GeneScanTM-500 LIZ^TM^ Size Standard (ThermoFisher Scientific, Waltham, MA, USA). The minimum detection threshold during fragment analysis was set at 150 relative fluorescence units (RFU) using OSIRIS software version 2.16 (https://www.ncbi.nlm.nih.gov/osiris/, accessed on 2 February 2023).

#### 2.4.2. Statistical Analyses

Based on the number of detected alleles, expected and observed heterozygosity (HE, HO), and allele frequencies, polymorphism information content (PIC) at each locus was determined [42]. To access the statistical confidence for individual identification, probability of identity (P_ID_) was calculated using GenAlEx v6.5 [43,44], which represents the probability that two individuals randomly selected from a population will have the same genotype at multiple loci [44,45]. The theoretical expected P_ID_ was computed for each locus with at least two alleles using allele frequencies from a population sample and the following equation: PI = 2 × [∑(pi^2^)^2^] − ∑(pi)^4^, where pi is the frequency of the ith allele.

### 2.5. Allele Sequencing of Polymorphic Microsatellites

Sanger-sequencing was applied to determine whether the targeted microsatellite locus was amplified in fallow deer samples. One homozygous allele per polymorphic locus was amplified with locus-specific primers without fluorescent labeling (Table S8). The amplification conditions were used as described in Section 2.3, with each representative allele amplified independently in singleplex reaction. Amplification products were purified using GenElute^TM^ PCR Clean-Up Kit (Sigma–Aldrich, St. Louis, MO, USA). Both DNA strands were sequenced using the BigDye^®^ Terminator v.1.1 Cycle Sequencing Kit (Thermo Fisher Scientific, Waltham, MA, USA) following the manufacturer’s recommendations. For sequence detection, an ABI Prism 3130XL Genetic Analyzer (Applied Biosystems, Waltham, MA, USA) was used, according to the manufacturer’s guidelines. Sequence analyses were performed using Sequencing Analysis Software 5.1 (Applied Biosystems, Waltham, MA, USA), and the sequences were aligned using Sequencher^TM^ 4.1.2 software (Gene Codes Corp, Ann Arbor, MI, USA). The resulting consensus sequences for each allele within a locus were used to determine the sequence, including the repeating units and flanking sequences.

## 3. Results

### 3.1. Marker Selection, Primer Design and Redesign, PCR Optimization

A total of 107 markers previously described as tetrameric microsatellites were selected, with 94 obtained from previous publications and an additional 13 markers obtained from the NCBI database (Table S1). During the systematic screening of the accession numbers for these markers available in the nucleotide database, it was discovered that several of them appeared in publications under different names, resulting in duplicates (OheF = C143, OheI = C180, OheK = C217, OheM = C273) (Table S2). Only one marker was used from each set of duplicates, namely OheF, OheI, OheK, and OheM, which were selected. Further, during the sequence alignment procedure, three additional duplicates were identified using BLAST: Capcap36 = T107, C02 = C36, and T268 = T530. The optimally functional marker tested using PCR was selected for further studies: T107, C02, and T268. In addition, while checking the primer sequences of markers not available in the database, it was noticed that two markers (ApoV144 and ApoV146) from the same publication shared the same primer pairs. Therefore, only one marker (ApoV146) was used during the research.

**Figure 1:**
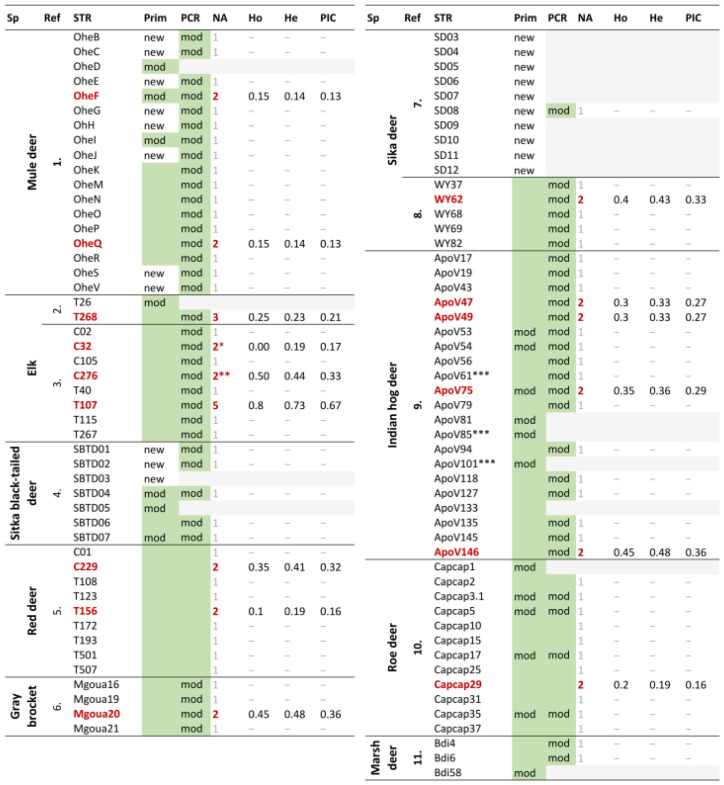
Details of the 99 tetrameric markers chosen on 20 fallow deer samples. Sp: species, Ref: reference of the original article, Prim: primers used (green background: original primers, mod: redesigned primers, new: designed primers), * data derived from 19 samples, ** data derived from 18 samples. . *** primers can be found in the NCBI database, not in the source publication. PCR—  : original PCR protocol, mod: modified PCR protocol,   : did not amplify. Polymorph marker names and their number of alleles are in red. References: 1.: Jones et al., 2000 [21]; 2.: Jones et al., 2001 [22]; 3.: Meredith et al., 2005 [23]; 4.: Brinkman et al., 2010 [24]; 5.: Szabolcsi et al., 2014 [25]; 6.: Caparroz et al., 2015 [26]; 7.: Accession numbers JN643715-JN643722, JN563734-JN563735; 8.: Yang et al., 2018 [27]; 9.: Hill et al., 2021 [28]; 10.: Morf et al., 2021 [29]; 11.: Wolfenson et al., 2022 [30].

After excluding duplicates, 99 unique microsatellites remained, requiring primer design in 21 cases due to the unavailability of primer sequences (Figure 1). For some “Ohe” markers, primers were designed because the marker selection process concluded before the currently available primer sequences were published. In the preliminary PCR testing, 33 markers failed to amplify a suitable amplicon using the original or designed primers; even after gradient-PCR optimization. In 26 cases, no PCR product was generated (OheD, OheF; OheI; T26; SBTD: 01, 03, 05; SD: 03–12; ApoV: 81, 85, 101, 133; Capcap: 1, 3.1, 5, 17, 35). Three markers produced very weak bands on the agarose gel (ApoV: 53, 54, 75), while four markers showed by-products (OheS, SBTD04, SBTD07, and Bdi58).

Primers were redesigned for 32 markers that failed to amplify a suitable amplicon. Therefore, at the end of the optimization process, 80 microsatellites out of the 99 originally selected loci showed detectable and apparently specific PCR products on the agarose gel (Figure 1). The sequence of the ApoV133 marker was not available in the NCBI database, so primer redesign was not possible. To maximize the probability of success for the loci with redesigned primers, we tested various combinations of the original or first-designed primers and the redesigned forward and reverse primers. The successful combinations are provided in the supplementary material (Table S9). Sixteen STRs were successfully amplified using their original protocols, and sixty-four markers were amplified using modified PCR protocols.

### 3.2. Success Rate and Characteristics of Amplified PCR Product

The detection of 80 functional markers on 20 fallow deer samples using capillary electrophoresis revealed that some markers were not successfully amplified in all samples, even after repeated attempts. Six markers (OheS, C32, C105, WY68, WY82, SD08) did not yield a PCR product in one sample, C276 in two samples, and Capcap37 in five samples (Table S10). Based on the patterns of the electropherograms, five markers were excluded from further analysis (Figure S2). Marker SBTD07 showed a “ladder-like” pattern with two-base-pair increments, while the Capcap2 microsatellite mostly exhibited a “ladder-like” pattern with one-base-pair increments, and some samples showed a significantly shorter (about 30 bp) product at this locus. In most samples tested for the WY82 marker, two peaks were detected consistently with the same length, which did not provide reliable results during repeated testing. For the ApoV43 marker, three peaks of the same length were visible in all samples. The WY62 marker exhibited a pattern typical of microsatellites with dimer units. Although ApoV49 and ApoV75 microsatellites exhibited a “ladder-like” pattern around their highest specific peaks with one-base-pair increments, they were included in further statistical analyses (Figure S3). The other 73 markers displayed clearly detectable peaks. Out of the 80 markers, 15 showed the characteristics of polymorphic microsatellites, and 14 of these were assumed to be tetrameric and were used for further analyses (Table 1, Figure S3).

### 3.3. Genotyping and Basic Statistical Values

Twenty samples were genotyped on the 14 microsatellites, and none of them exhibited the same genetic profile (Table S11). The number of alleles ranged from two to five, with 12 out of the 14 markers being biallelic (Figure 1). The frequency of the most common allele per locus varied from 0.425 to 0.925 (Table S11). Expected and observed heterozygosity ranged between 0.14 and 0.73 (mean 0.32) and 0.00 and 0.80 (mean 0.28), respectively, while the polymorphic information content (PIC) was between 0.13 and 0.67 (mean 0.28) (Figure 1, Table S11). Upon examining deviations from Hardy–Weinberg Equilibrium, T156 showed significant deviation (*p* < 0.05), while T32 displayed highly significant deviations (*p* < 0.001) (Table S12). The calculated PI value for the 14 markers was 5.2 × 10^−5^.

### 3.4. Allele Sequencing

One homozygous allele per polymorphic marker was sequenced to verify the STR structures (Acc. numbers: OQ981365-OQ981367, OQ981369-OQ981380). Fourteen markers exhibited tetranucleotide repeat motifs. However, unlike the source species, the WY62 microsatellite displayed a dinucleotide repeat. It has been established that the single base pair ladder-like patterns observed in the ApoV49 and ApoV75 markers are the result of mononucleotide repeats of 10 and 14 bp in length, respectively, within their sequences.

## 4. Discussion

Despite the wide distribution of the species and the availability of samples, genetic studies on fallow deer are quite limited [3]. In this study, we aimed to identify polymorphic markers for fallow deer by testing 99 unique tetrameric microsatellite markers. These markers have been previously described to have various alleles in different deer species. Tetrameric microsatellites offer advantages such as more reliable allele detection and better inter-laboratory comparability compared with their dimeric and trimeric counterparts [21,46]. However, during our own study, we discovered that certain markers originally classified as tetrameric were actually dimeric in fallow deer (e.g., WY62). Stuttering, which refers to the formation of by-products, can complicate accurate genotyping and raise questions about the results, particularly in cases involving sample mixture or contamination [47]. Nevertheless, tetrameric markers can be valuable in conservation, parentage studies, and forensic investigations where determining the correct genetic profile is crucial, especially when dealing with varying sample quality and quantity. It is important to note that tetrameric microsatellites tend to be less polymorphic than trimeric or dimeric ones [21,25], and they are less abundant in the genome [21].

Out of the 99 markers selected, 19 produced non-specific or inappropriate PCR products, which can be attributed to various factors. Firstly, mutations in the target species can inhibit proper primer binding, a well-known challenge that restricts the cross-species application of markers developed for other species [48,49]. Secondly, the adapter sequence used for labeling forward primers with fluorophores, which offers a cost-effective method for testing numerous loci, can significantly interfere with amplification. Luttman et al. [50] have previously highlighted the potential primer dimerization caused by the added universal primer sequence. During our research, we also encountered other issues unrelated to primary dimers (Appendix A). Overall, while the end-labeling technique provides a cost-effective option for testing a large number of loci, it may pose challenges in assembling multiplex panels.

Among the 80 functional STRs tested in our study, 15 markers (19%) were polymorphic in 20 fallow deer samples. When Poetsch et al. (2001) tested a set of twelve dimeric STR markers on red deer, roe deer, and fallow deer, they found that while the former two species were polymorphic at ten and nine loci, respectively, the fallow deer was polymorphic at only seven [34]. Based on the testing of 142 cross-specific di-, tri-, and tetranucleotide markers on hog deer, 18 STRs showed some level of polymorphism [28]. Regarding allelic richness, our polymorphic markers contained two alleles, except for loci T268 and T107, which had three and five alleles, respectively. In this study, the average allele number was 2.29 for fourteen tetrameric loci, which is close to the value of panels using different numbers of dimeric microsatellites in fallow deer (2.29–3.65) (Table A1). Comparing our average heterozygosity results (Ho = 0.31 and He = 0.33) with previous publications on fallow deer (Table A1), we found these data to be slightly higher using a dinucleotide microsatellite panel (Ho = 0.36–0.45 and He = 0.40–0.44). Table A1 also shows that in many cases, a higher allelic number did not come with better heterozygosity, indicating that even markers with fewer alleles can prove to be useful. However, the range of polymorphic information content in our study (from 0.13 to 0.67) was better than the one using dimeric microsatellites (between 0.06 and 0.59). The average PIC was better using the dimeric panel (0.36) than the same value using our tetrameric set (0.28) [51].

Altogether, based on the calculated basic statistical values, dimeric markers can perform better to some degree than their tetrameric counterparts. Comparing the parameters of our selected tetrameric markers with the values of the same microsatellites in different deer species shows that the performance of the markers is fundamentally influenced by the characteristics of the studied population (Table A2). Therefore, population genetic indicators from different studies might be significantly influenced by differences in sample size, fraction of males and females, marker number, and sets of markers used, reducing the comparability between studies [32].

Interpreting the calculated combined PI in our study (5.2 × 10^−5^), the result shows that out of 100,000 individuals, 5.2 may have the same genotype using the selected set of 14 tetrameric STRs. Considering the fallow deer herd in Hungary, which is approximately 40,000 individuals, this means that two individuals may have the same genetic profile. This conclusion is also supported by the genotypes of the 20 fallow deer we examined, which were all unique. The deviation of the two markers (C32, T156) from the Hardy–Weinberg equilibrium was due to the small number of samples, which may also cause the low number of alleles per marker. Two marker groups from Jones et al. (2000) [21] and from Szabolcsi et al. (2014) [25] examined in this study were also tested on 27 other fallow deer samples, showing a third allele in marker OheQ [52]. This suggests that several other markers may exhibit similar patterns.

Our results indicate low genetic polymorphism in the Hungarian fallow deer population, which is in accordance with previous studies conducted on the species using different types of markers [14,15,17,18,19,20,34,51,53,54,55]. All of them showed overall low genetic diversity within [17] and even among populations, which can be explained by a combination of natural and anthropogenic processes [14]. The species’ past extinction from its main European distribution during the Pleistocene [56], the subsequent human-mediated reintroduction and translocations throughout history from even before the Romans to recent times [14,57,58,59,60,61,62], and the polygynous mating system [19,55] are all suspected to be behind the current lack of genetic diversity.

However, the current set of selected markers has room for improvement, such as using next-generation sequencing to find other tetrameric loci. Nonetheless, its usefulness as a basis for further development is indisputable. Once we have a statistically robust set of markers, we plan to proceed with a validation process. It is important to note that different populations may exhibit polymorphism in different markers, as demonstrated by Baker et al. (2017) [14]. Therefore, it could be worthwhile to test markers that were monomorphic in our samples when conducting research on other populations.

## 5. Conclusions

Tetrameric microsatellites, while less abundant in the genome and usually less polymorphic compared with dimeric or trimeric microsatellites, can still be useful in genetic studies, especially when correct and reliable genotyping is essential. The set of 14 polymorphic tetranucleotide loci tested in fallow deer in this study performed almost similarly to the panels using dimeric loci found in many other publications.

## Figures and Tables

**Table 1:** The 14 selected polymorphic fallow-deer-specific tetrameric microsatellite markers.

Marker	Tail	Size (bp)	Forward Primer	Reverse Primer	PCR Protocol
OheF	B	199–211	CAGGCGATCAAGAAATGTGG	GTGGCTTCTGGATGGAGAAC	32 × (94 °C 30 s, 56 °C 30 s, 72 °C 30 s) + 72 °C 20 m
OheQ	D	248–264	AATGTGTCAGTGAAGGTCTTC	ATCCAGGCAACCATCTAG	
C229	A	117–125	TTATTCATCCACCCATCCATCACCA	GGCACATGCTCATAAGTGAAGGGA	29 × (94 °C 40 s, 61 °C 40 s, 72 °C 60 s) + 60 °C 60 m
T156	C	135–148	CCTGGCCTGTGTCTTGAATTGAAC	GGCGATGAATACCCAGTCTTGTCT	29 × (94 °C 40 s, 63 °C 40 s, 72 °C 60 s) + 60 °C 60 m
Capcap29	C	199–203	AAGCCCATGACCTGAAACCAA	GCTTCCAGCAGGAGGGTATAT	5 × (94 °C 30 s, 62 °C 90 s, 72 °C 90 s)
					5 × (94 °C 30 s, 58 °C 90 s, 72 °C 90 s)
					5 × (94 °C 30 s, 55 °C 90 s, 72 °C 90 s)
					20 × (94 °C 30 s, 50 °C 90 s, 72 °C 90 s) + 72 °C 10 m
ApoV47	A	329–333	TGCTCATTCTAGGGTCAGGC	AGGTCTTCTGCATTGTAGGC	32 × (94 °C 30 s, 64 °C 30 s, 72 °C 60 s) + 60 °C 30 m
ApoV49	B	408–412	ACTATGGGATGTGACCGTGG	ACAGGAATCTTGTTGACTCTGC	32 × (94 °C 30 s, 56 °C 30 s, 72 °C 60 s) + 60 °C 30 m
ApoV146	D	143–148	GGGCCCTCAATTCTCTTCC	GGAGACATCACATTCCCTGAC	32 × (94 °C 30 s, 58 °C 30 s, 72 °C 60 s) + 60 °C 30 m
Mgoua20	B	193–197	ACAACTGGAGAAAACCCTTGTG	AGCCTTTAGAGATGTTCTGTTTGG	15 × (94 °C 30 s, 55 °C 60 s, 72 °C 40 s) 20 × (94 °C 30 s, 50 °C 60 s, 72 °C 40 s) + 72 °C 20 m
T107	D	283–308	ACATCCGTTCAGGTGTGA	CCAGAGGTAAGATAAATGGTGA	
T268	B	224–240	ATTCCCTTCTCCAGTGTATG	GATGATAACAGCTCAACAGATC	
C32	A	285–289	ACAACTGTGTGAGCCAATAC	AGCAAGTGAAGAAGAATGTTC	15 × (94 °C 30 s, 60 °C 60 s, 72 °C 40 s)
					20 × (94 °C 30 s, 55 °C 60 s, 72 °C 40 s) + 72 °C 20 m
C276	A	376–380	AAACAGAACATTCACCAGAAAC	TCCCAGACACACAGAACAA	32 × (94 °C 30 s, 63.3 °C 60 s, 72 °C 40 s) + 72 °C 20 m
ApoV75	A	328–336	TCGTTTTACATTCCTATCAGCAACG	GTTTCTTTACTGAGATGCCGACTCCCA	

bp: base pair; bold bases in the reverse primer sequence means a pigtail addition; PCR protocol: cycle number × (denaturation, annealing, and elongation temperatures and time) + final elongation temperature and time; s: second; m: minute.

## Data Availability

The data will be available from the corresponding author upon request.

## Supplementary Materials

The following are available online at https://www.mdpi.com/article/10.3390/ani13132083/s1, Figure S1: Distribution of collected fallow deer samples (n = 20) within Hungary; Figure S2: Markers excluded from further analysis based on the electrophoretic pattern; Figure S3: 14 polymorphic markers included in further statistical analysis based on their electrophoretic pattern; Table S1: Data consist of 107 cross-species tetranucleotide microsatellite markers, with an additional 3 markers used for preliminary in silico marker selection; Table S2: Same markers published or used under different names in separate articles; Table S3: Primers designed for 21 markers for which no published primer sequences were available; Table S4: Data of the four used universal tailed primers; Table S5: Forward primers with universal adapters and different fluorescent dyes, and the optimized PCR protocols used for amplification; Table S6: Redesigned primer pairs; Table S7: Parallel detection of 4–8 PCR products with different sizes or with different fluorescent labels using capillary electrophoresis; Table S8: Sanger-sequencing with locus-specific primers without fluorescent labeling; Table S9: Primer mixtures with successful amplifications; Table S10: Fallow deer samples not successfully amplified with certain markers; Table S11: Genotypes and statistical analysis of 20 fallow deer samples based on 14 polymorphic microsatellite markers; Table S12: Summary of Chi-Square Tests for Hardy–Weinberg Equilibrium on our markers.

## Appendix A

Universal primers have their own optimal annealing temperature, which must be taken into account when setting up the PCR protocol. However, the annealing temperatures of the primer mixtures containing the universal primer often did not correspond to the optimum of either the original or the universal primer and showed temperatures different from both annealing temperatures. Furthermore, the addition of the fluorescently labeled universal primer to the labeled forward primer completely changed again the annealing temperature optimum. Apart from this, many primers could not be detected by gel electrophoresis after PCR, either as primer dimers or as unused primers. This may be due to the invisible phenomenon of “primer clusters” [63]. The technique makes it difficult to efficiently design multiplexes or even primer pairs, since with the additional universal sequence, the forward and reverse primers often no longer have the same GC content.

## Appendix B

**Table A1:** Comparison of the statistical data of our marker set and dimer marker sets from previous articles.

	STR = 14; N = 20 [own study]			STR = 10; N = 52 [20]			STR = 20; N = 262 [55]		
	NA	Ho	He	NA	Ho	He	NA	Ho	He
average	2.29	0.31	0.33	2.40	0.45	0.44	3.65	0.42	0.44
median	2.00	0.30	0.33	2.00	0.49	0.45	4.00	0.41	0.44
min	2.00	0.00	0.14	2.00	0.21	0.23	2.00	0.16	0.20
max	5.00	0.80	0.73	4.00	0.56	0.58	7.00	0.81	0.73
	STR = 7; N = 22 [34]			STR = 9; N = 111 [53]			STR = 10; N = 14 (only HU) [14]		
	NA	Ho	He	NA	Ho	He	NA	Ho	He
average	2.29	0.36		3.56	0.37	0.41	2.67	0.40	0.40
median	2.00	0.41		3.00	0.38	0.38	2.00	0.38	0.46
min	2.00	0.09		2.00	0.06	0.06	2.00	0.07	0.07
max	3.00	0.64		5.00	0.68	0.67	4.00	0.79	0.69

STR: number of microsatellites used in the publication; N: sample size; NA: number of alleles; Ho: observed heterozygosity; He: expected heterozygosity. The best results in the comparison are highlighted in green, while the worse results are highlighted in red.

**Table A2:** Comparison of our polymorphic tetramer STRs in fallow deer and in other deer species.

	OheF				OheQ				C229					T156			Capcap29		
Species [ref]	FD	Elk [23]	Mule [31]	WT [31]	FD	Mule [21]	Mule [31]	WT [31]	FD	Elk [23]	Mule [31]	WT [31]	Red [25]	FD	Elk [23]	Red [25]	FD	Roe [29]	
N	20	30	556	587	20	602	556	587	20	30	556	587	100	20	43	100	20	513	
n	2	4	6	10	2	15	5	31	2	4	16	15	5	2	9	15	2	9	
PIC	0.13		0.36	0.75	0.13		0.85	0.93	0.32		0.64	0.79	0.45	0.16		0.89	0.16	0.72	
Ho	0.15	0.87	0.32	0.65	0.15		0.53	0.84	0.35	0.50	0.61	0.68	0.49	0.10	0.44	0.85	0.20	0.74	
He	0.14	0.69	0.38	0.78	0.14		0.86	0.93	0.41	0.69	0.68	0.81	0.50	0.19	0.68	0.90	0.19	0.76	
	C32		T107			ApoV47		T268		C276		ApoV49		Mgoua20		ApoV146		ApoV75	
Species [ref]	FD	Elk [23]	FD	Elk [23]	Roe [29]	FD	Hog [28]	FD	Elk [23]	FD	Elk [23]	FD	Hog [28]	FD	BB [26]	FD	Hog [28]	FD	Hog [28]
N	19	24	20	30	513	20	200 *	20	43	18	27	20	213	20	14	20	208	20	200 *
n	2	2	5	4	12	2	1	3	4	2	2	2	3	2	3	2	3	2	1
PIC	0.17		0.67		0.81	0.27		0.21		0.33		0.27	0.13	0.36		0.36	0.42	0.29	
Ho	0.00	0.37	0.80	0.37	0.80	0.30	0.00	0.25	0.53	0.50	0.12	0.30	0.00	0.45	0.54	0.45	0.13	0.35	0.00
He	0.19	0.32	0.73	0.49	0.83	0.33	0.00	0.23	0.71	0.44	0.12	0.33	0.14	0.48	0.42	0.48	0.53	0.36	0.00

FD: fallow deer, Elk: California elk, Mule: mule deer, WT: white-tailed deer, Red: red deer, Roe: roe deer, Hog: hog deer. N: sample size, n: number of alleles, PIC: polymorphism information content, Ho: observed heterozygosity, He: expected heterozygosity. *: tested on somewhere between 170 and 224 samples. The best results in the comparison are highlighted in green, while the worse results are highlighted in red.
