# Supplementary material for: A Selection of 14 Tetrameric Microsatellite Markers for Genetic Investigations in Fallow Deer (Dama dama)
Source: Animals (Basel). 2023 Jun 23;13(13):2083. doi: 10.3390/ani13132083 (PMC10339914; doi:10.3390/ani13132083)
Supplement: Supplementary file 1 [file animals-13-02083-s001.zip › animals-2435056-supplementary.pdf]

Table S1. Data of the 107 plus additional 3 cross-species tetranucleotide microsatellite markers for preliminary *in silico* marker selection. (~~OheA~~, ~~OheL~~ and ~~OheT~~ markers were previously eliminated from the study as they were identical with C01, C229 and OheP markers, respectively [52]). Eliminated duplicates are with gray background.

Species	Tetrameric microsatellites	Reference
Mule deer (*Odocoileus hemionus*)	~~OheA~~, OheB, OheC, OheD, OheE, OheF, OheG, OheH, OheI, OheJ, OheK, ~~OheL~~, OheM, OheN, OheO, OheP, OheQ, OheR, OheS, ~~OheT~~, OheV	[21]
Elk (*Cervus canadensis*)	C217, T26, T268, T530	[22]
	C02, C32, C36, C105, C143, C180, C273, C276, T40, T107, T115, T267	[23]
Sitka black-tailed deer (*Odocoileus hemionus sitkensis*)	SBTD01, SBTD02, SBTD03, SBTD04, SBTD05, SBTD06, SBTD07	[24]
Red deer (*Cervus elaphus*)	C01, C229, T108, T123, T156, T172, T193, T501, T507	[25]
Brown brocket (*Mazama gouazoupira*)	Mgoua16, Mgoua19, Mgoua20, Mgoua21	[26]
Sika deer (*Cervus nippon*)	WY37, WY62, WY68, WY69, WY82	[27]
	SD03, SD04, SD05, SD06, SD07, SD08, SD09, SD10, SD11, SD12	JN643715-22, JN563734-35
Indian hog deer (*Axis porcinus*)	ApoV17, ApoV19, ApoV43, ApoV47, ApoV49, ApoV53, ApoV54, ApoV56, ApoV61, ApoV75, ApoV79, ApoV81, ApoV85, ApoV94, ApoV101, ApoV118, ApoV127, ApoV133, ApoV135, ApoV144, ApoV145, ApoV146	[28]
Roe deer (*Capreolus capreolus*)	Capcap1, Capcap2, Capcap3.1, Capcap5, Capcap10, Capcap15, Capcap17, Capcap25, Capcap29, Capcap31, Capcap35, Capcap36, Capcap37	[29]
Marsh deer (*Blastocerus dichotomus*)	Bdi4, Bdi6, Bdi58	[30]

Table S2. Same markers published or used under different names in separate articles.

Zorkoczy et al. 2023	Jones et al. 2000 [21]	Jones et al. 2002 [22]	Meredith et al. 2005 [23]	Brinkman et al. 2010 [24]	Hamlin et al. 2021 [31]
**C01**	A		C01		
**OheB**	B				OheC10-B
**OheC**	C				OheC50-C
**OheD**	D			C89	OheC89-D
**OheE**	E				
**OheF**	F		C143		OheC143-F
**OheG**	G				
**OheH**	H				OheC165-H
**OheI**	I		C180		
**OheJ**	J				OheC186-J
**OheK**	K	C217		C217	OheC217-K
**C229**	L		C229		OheC229a-L
**OheM**	M		C273	C273	OheC273-M
**OheN**	N			T27	OheT27r-N
**OheO**	O			T159S	OheT159-O
**OheP**	P			T7	OheT7-P
**OheQ**	Q			T32	OheT32-Q
**OheR**	R			C106	
**OheS**	S				
**OheT**	T				
**OheV**	V				OheT256-V

Table S3. Designed primers for 21 markers for which no published primer sequences were available.

Marker	Primer sequence (5'-3')	Marker	Primer sequence (5'-3')
OheB	F: GTCTGCTCATCCACCATCTA	SD03	F: GAGCCTGGCCAACATGGTA
	R: CTCTGTCTGCCTTCTCAAGT		R: CTGCCACCACAACCAGCTAA
OheC	F: TTCCATCCACTCATCATTCT	SD04	F: TTCTCCACCACTCCTTCT
	R: ACATATTGGAGGCATGTAGG		R: AGTAATAGATGTGCGGTCTC
OheE	F: AGACAGGGTTCCAATGAGAC	SD05	F: GGTGACAGTGCAAGACTC
	R: CAGCCTTCCTGGACTAGAGA		R: CGTTTTCCAGCACTATTC
OheG	F: AGAGTTAAGTGCAGCCTAAG	SD06	F: TCCTGGTGTGAGCCACAGT
	R: CTGTGTTCATGGACCACACT		R: CTGAGACATGCCACTGCACT
OheH	F: CTTGCTGCCATTGCCAGATA	SD07	F: GGCAACATAGCGAGAACT
	R: TCCCAGAGGGCAGATGTCTAT		R: CCATGGAGGTAAGACTCTTC
OheJ	F: ATCACAGTTGCCAAGACGAC	SD08	F: CACCGCCCCTGGCCTTTT
	R: CGGATGGATGAGTAGGTTGA		R: GAGGTTGCAGTCAGCCGAGATTG
OheS	F: AACCTGGCAAGTTACAGTCC	SD09	F: GCTGCTCAAGAAAGTTACC
	R: TCAGGCATGTGGAGCAACAA		R: GTAATTGTAGGGCCAGTT
OheV	F: TCAGGCAGGTAGTAAGTGTC	SD10	F: GTGGCCTCTGGCTTCATT
	R: GAGCACAGGACCTGATACTT		R: GCCTGGCAACATAACAAGAC
SBTD01	F: TCACTCACCCACTCATCT	SD11	F: GTTCAACAGAGGAAGCTGTA
	R: TGGATGGTTGGATATGTAG		R: TCTAGGCAACAGAGTGAGAC
SBTD02	F: CTCTGCTGACCTGTTATCTC	SD12	F: GCCAAGCTCTTCTTCATCCT
	R: ATGGAAGAAAGGATGTCTG		R: GCTGCGGTGGAGCTATTAT
SBTD03	F: GCCACCTCTTCTTAATATC		
	R: ATGGTAGGGACCTAACAG		

Table S4. Data of the four used universal tailed primers.

Universal primers	Tail sequence (5'-3')	Fluorescent dye	Length (base pair)	Melting temperature (Tm)
Tail **A**	GCCTCCCTCGCGCCA	FAM	15	63°C
Tail **B**	GCCTTGCCAGCCCGC	VIC	15	57°C
Tail **C**	CAGGACCAGGCTACCGTG	NED	18	59°C
Tail **D**	CGGAGAGCCGAGAGGTG	PET	17	59°C

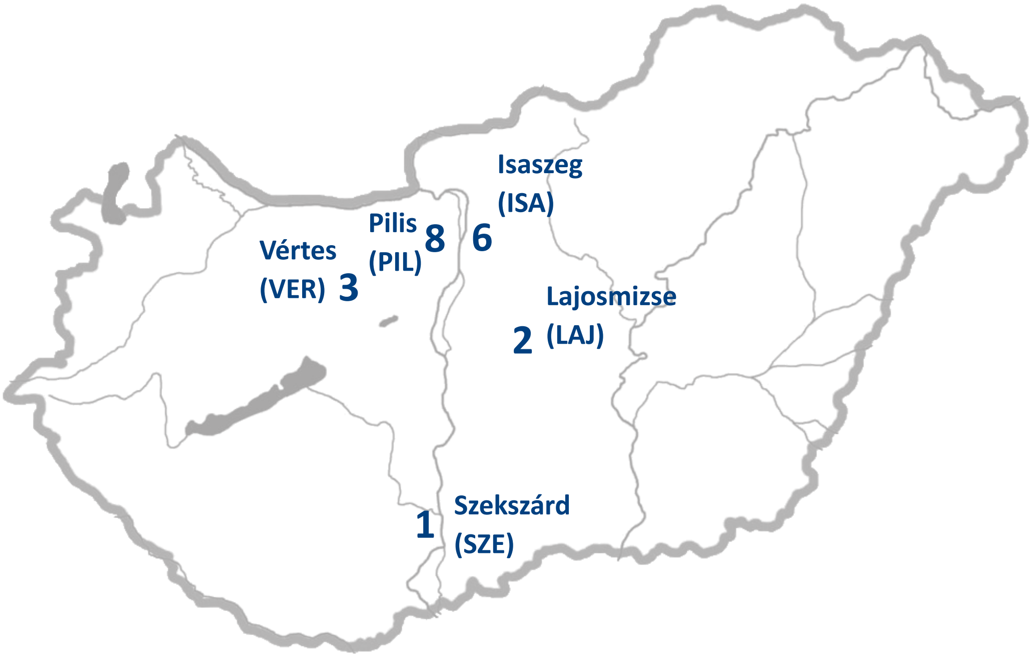

Figure S1. Distribution of collected fallow deer samples within Hungary from (n=20). The original name of the location, the code name in brackets and the number of samples from that location.

Table S5. Forward primers with universal adapters and different fluorescent dyes, and the optimized PCR protocols used for amplification. **A**: FAM, **B**: HEX, **C**: NED, **D**: PET.

Locus	Universal primer	PCR protocol	Locus	Universal primer	PCR protocol
C229	Tail **A**		ApoV17	Tail **A**	
T108	Tail **B**	**[25]**	ApoV49	Tail **B**	**3.**
T123	Tail **D**		ApoV127	Tail **D**	
T193	Tail **C**		ApoV47	Tail **A**	**4.**
C01	Tail **B**		ApoV56	Tail **B**	
T156	Tail **B**		C02	Tail **C**	
T172	Tail **C**	**[25]**	T107	Tail **D**	
T501	Tail **A**		T268	Tail **B**	
T507	Tail **D**		SBT0D6	Tail **B**	
CapCap2	Tail **A**		Mgou16	Tail **C**	**5.**
CapCap10	Tail **A**		Mgou19	Tail **A**	
CapCap15	Tail **C**		Mgou20	Tail **B**	
CapCap25	Tail **B**	**[29]**	Mgou21	Tail **C**	
CapCap29	Tail **C**		WY37	Tail **B**	
CapCap31	Tail **B**		WY69	Tail **C**	**6.**
CapCap37	Tail **C**		C32	Tail **A**	
OheB	Tail **A**		CapCap3.1	Tail **B**	
OheC	Tail **D**		CapCap5	Tail **D**	
OheE	Tail **C**		CapCap17	Tail **A**	
OheF	Tail **B**		CapCap35	Tail **B**	**7.**
OheG	Tail **B**		OheS	Tail **B**	
OheH	Tail **B**		C105	Tail **C**	
OheI	Tail **A**		SBT0D4	Tail **A**	
OheJ	Tail **A**	**1.**	T267	Tail **D**	
OheK	Tail **D**		SD08	Tail **B**	
OheM	Tail **A**		SBTD01	Tail **B**	**8.**
OheN	Tail **A**		SBTD02	Tail **C**	
OheO	Tail **C**		WY68	Tail **D**	
OheP	Tail **C**		WY82	Tail **D**	
OheQ	Tail **D**		C276	Tail **A**	
OheR	Tail **C**		T40	Tail **A**	
OheV	Tail **C**		T115	Tail **A**	
ApoV19	Tail **A**		SBTD07	Tail **A**	
ApoV43	Tail **A**		WY62	Tail **A**	**9.**
ApoV79	Tail **B**		ApoV53	Tail **D**	
ApoV94	Tail **D**	**2.**	ApoV54	Tail **B**	
ApoV118	Tail **D**		ApoV61	Tail **D**	
ApoV135	Tail **B**		ApoV75	Tail **A**	
ApoV145	Tail **D**		Bdi04	Tail **A**	
ApoV146	Tail **D**		Bdi06	Tail **B**	

PCR prot.	Inicial denat.	Cycle nu.	Denat.	Anneal.	Elong.	Cycle nu.	Denat.	Anneal.	Elong.	Final elong.
**1.**	1 min 94°C	32	30 sec 94°C	30 sec 56°C	30 sec 72°C					20 min 72°C
**2.**		32	30 sec 94°C	30 sec 58°C	1 min 72°C					30 min 60°C

**3.**	32	30 sec 94°C	30 sec 56°C	1 min 72°C					30 min 60°C
**4.**	32	30 sec 94°C	30 sec 64°C	1 min 72°C					30 min 60°C
**5.**	15	30 sec 94°C	1 min 55°C	40 sec 72°C	20	30 sec 94°C	1 min 50°C	40 sec 72°C	20 min 72°C
**6.**	15	30 sec 94°C	1 min 60°C	40 sec 72°C	20	30 sec 94°C	1 min 55°C	40 sec 72°C	20 min 72°C
**7.**	32	30 sec 94°C	1 min 56°C	40 sec 72°C					20 min 72°C
**8.**	32	30 sec 94°C	1 min 58.8°C	40 sec 72°C					20 min 72°C
**9.**	32	30 sec 94°C	1 min 63,3°C	40 sec 72°C					20 min 72°C

Table S6. Redesigned primer pairs. Non-redesigned forward or reverse primers are in italic.

Marker	Primer sequence (5'-3')	Marker	Primer sequence (5'-3')
SD03	F: TAGGTCGGGAGTTCGAGACG	Capcap1	F: GGACAGGACCTTCTGAACTA
	R: TGCCACCACAACCAGCTAAT		R: CCTCTCGCTTCTTCCTACTA
SD04	F: CACCACTCCTTCTCCCCTGA	Capcap3.1	F: TCCTTGTCTATCTCTCTGTCTGTC
	R: GAGACCGCACATCTATTACTCTT		R: GAACCAGCAGATAGGCAGAT
SD05	F: CCAGGTGACAGTGCAAGACT	Capcap5	F: CCAGCTCTCATACAACAC
	R: TCGTTTTCCAGCACTATTCTTTC		R: *ACGTGGGTTAATGGTTCAAGGT*
SD06	F: ACAGTGCCCAGCCTTATTCC	Capcap17	F: GGTTGGTACACGGAGAGT
	R: TGAGCTGAGACATGCCACTG		R: CTAGAGGCTCTGTGTATG
SD07	F: CCTGGGCAACATAGCGAGAA	Capcap35	F: CCAAACACCCTGAATCCTCT
	R: *CCATGGAGGTAAGACTCTTC*		R: TCTTGAATCAGGGAATGGAGACT
SD08	F: ACAGGTATGAGCCACCGC	OheD	F: *AAGGAACAGAGCCTCGTCTT*
	R: TGGGGACAGAGCGAGATTCA		R: GTCTGACACCTGGGGTTCTG
SD09	F: *GCTGCTCAAGAAAGTTACC*	OheF	F: CAGGCGATCAAGAAATGTGG
	R: GCCAGTTCTGGGACCTTTCT		R: GTGGCTTCTGGATGGAGAAC
SD10	F: TCTATGTTTGTGGCCTCTGG	OheI	F: ACATGGTGGGCATTCAGTAG
	R: TGGCAACATAACAAGACATTTTCT		R: CATCAGGGTTCTCTGAAGAC
SD11	F: TACAGCCCGCCAAACTTCTT	OheS	F: *AACCTGGCAAGTTACAGTCC*
	R: GGCAACAGAGTGAGACCCTT		R: CCACATGCCTGACACTTCTA
SD12	F: AGGGCTGCTAGGGAGTTAGG	Bdi58	F: TCTTCATGGGGGGTATGT
	R: GGCTGCGGTGGAGCTATTAT		R: TGGTGGCAGTGAAACTTG
SBTD01	F: TCCAACCACCCACATATGCA	ApoV53	F: ACATGTGACGAGCAGCTTCA
	R: TGGATGGTTGGATATGTAGGCA		R: *TGTGATCCAGGTGAGAGCAG*
SBTD03	F: TCTTGCCACCTCTTCTTAATATCT	ApoV54	F: AGGGCAGCTGATACCCATTT
	R: AGGGCAGAAATGGTAGGGAC		R: ACACACCTAAGTCCCAAGCA
SBTD04	F: CTGCCTGCTCCCTCTAACTC	ApoV75	F: *TCGTTTTACATTCCTATCAGCAACG*
	R: GGCCTTGTCCCTGAGATTGG		R: TACTGAGATGCCGACTCCCA
SBTD05	F: CATGACATGTACTAAGGGGCA	ApoV81	F: CGCACACACACACATACACG
	R: GGAGATCCAAGTACACACATGT		R: AACACGATGGAAGCCTCTGG
SBTD07	F: TCCAGAACTGTGTGTGAGCC	ApoV85	F: *ACAGCAGTTTCCCAAATTTGTG*
	R: ACTTGTATCAGTCTGGGTTCGT		R: TCTTCCTGCCTGACTCTGGA
T26	F: CCACCCATGCAGATGCTACT	ApoV101	F: *CTGGTTTCACTTTGCCGGTC*
	R: GCTGTCAAGGCAACATTCTC		R: GAGTCACAATCCTCCTGAAGGTCTC

Table S7. Parallel detection of 4-8 PCR products with different sizes or with different fluorescent labels using capillary electrophoresis. Blue background means FAM-6 fluorescent dye, green background means HEX fluorescent dye, yellow background means NED fluorescent dye, red background means PET fluorescent dye,

Mix	Marker	Mix	Marker	Mix	Marker	Mix	Marker	Mix	Marker	Mix	Marker
mix01	OheB	mix04	C32	mix06	OheM	mix08	C229	mix11	ApoV19	mix13	SBTD04
	OheN		T115		OheH		T108		Mgoua19		ApoV47
	OheF		WY37		OheP		T193		WY62		ApoV56
	OheO		T268		OheV		T123		Mgoua20		SD08
	OheC		Mgoua21		OheQ	mix09	T501		ApoV135		SBTD02
mix02	Capcap2		T267	mix07	Apov17		C01		WY69		Mgoua16
	Capcap29		WY82		T40		T156		ApoV146		ApoV61
	Capcap37	mix05	OheI		C276		T172	mix12	ApoV43		T107
	SBTD01		OheJ		OheS		T507		SBTD06	mix14	Bdi4
mix03	Capcap10		OheG		ApoV49	mix10	Capcap17		ApoV79		SBTD07
	Capcap15		OheE		C02		Capcap3.1		C105		ApoV75
	Capcap25		OheR		ApoV127		Capcap35		ApoV94		Bdi6
	Capcap31		OheK		WY68		Capcap5		ApoV145		ApoV54
									ApoV118		ApoV53

Table S8. Sanger-sequencing with locus specific primers without fluorescent labelling. Pigtail is in bold.

Marker	Primer sequence (5'-3')	Marker	Primer sequence (5'-3')
OheF	F: CAGGCGATCAAGAAATGTGG	C276	F: AAACAGAACATTCACCAGAAAC
	R: GTGGCTTCTGGATGGAGAAC		R: TCCCAGACACACAGAACAA
OheQ	F: AATGTGTCAGTGAAGGTCTTC	ApoV49	F: ACTATGGGATGTGACCGTGG
	R: ATCCAGGCAACCATCTAG		R: ACAGGAATCTTGTTGACTCTGC
C229	F: TTATTCATCCACCCATCCATCACCA	WY62	F: TGCAGCTATGCTGGAGAGTC
	R: GGCACATGCTCATAAGTGAAGGGA		R: GCCCATGCATAAGATCCTTC
T156	F: CCTGGCCTGTGTCTTGAATTGAAC	Mgou20	F: ACAACTGGAGAAAACCCTTGTG
	R: GGCGATGAATACCCAGTCTTGTCT		R: AGCCTTTAGAGATGTTCTGTTTGG
Capcap29	F: AAGCCCATGACCTGAAACCAA	ApoV146	F: GGGCCCTCAATTCTCTTCC
	R: GCTTCCAGCAGGAGGGTATAT		R: GGAGACATCACATTCCCTGAC
T107	F: ACATCCGTTCAGGTGTGA	C32	F: ACAACTGTGTGAGCCAATAC
	R: CCAGAGGTAAGATAAATGGTGA		R: AGCAAGTGAAGAAGAATGTTC
ApoV47	F: TGCTCATTCTAGGGTCAGGC	ApoV75	F: TCGTTTTACATTCCTATCAGCAACG
	R: AGGTCTTCTGCATTGTAGGC		R: **GTTTCTT**TACTGAGATGCCGACTCCCA
T268	F: ATTCCCTTCTCCAGTGTATG		
	R: GATGATAACAGCTCAACAGATC		

Table S9. Primer mixtures with successful amplifications. Non-redesigned forward or reverse primers are in italic, pigtail is in bold.

Marker	Primer sequence (5'-3')	Marker	Primer sequence (5'-3')
OheF	F: CAGGCGATCAAGAAATGTGG	SBTD01	F: TCCAACCACCCACATATGCA
	R: GTGGCTTCTGGATGGAGAAC		R: TGGATGGTTGGATATGTAGGCA
OheI	F: ACATGGTGGGCATTCAGTAG	SBTD04	F: CTGCCTGCTCCCTCTAACTC
	R: CATCAGGGTTCTCTGAAGAC		R: GGCCTTGTCCCTGAGATTGG
OheS	F: *AACCTGGCAAGTTACAGTCC*	SBTD07	F: TCCAGAACTGTGTGTGAGCC
	R: CCACATGCCTGACACTTCTA		R: ACTTGTATCAGTCTGGGTTCGT
Capcap3.1	F: *TGTCTTTCCTTGTCTATCTCTCTGT*	ApoV53	F: ACATGTGACGAGCAGCTTCA
	R: GAACCAGCAGATAGGCAGAT		R: *TGTGATCCAGGTGAGAGCAG*
Capcap5	F: CCAGCTCTCATACAACAC	ApoV54	F: AGGGCAGCTGATACCCATTT
	R: *ACGTGGGTTAATGGTTCAAGGT*		R: ACACACCTAAGTCCCAAGCA
Capcap17	F: GGTTGGTACACGGAGAGT	ApoV75	F: *TCGTTTTACATTCCTATCAGCAACG*
	R: *GGAAGTCCCACTAGAGGCTCT*		R: TACTGAGATGCCGACTCCCA
Capcap35	F: CCAAACACCCTGAATCCTCT	SD08	F: *CACCGCCCCTGGCCTTTT*
	R: ***GTTT**CTCCCTCGGGATAATCAAGTATT*		R: TGGGGACAGAGCGAGATTCA

Table S10. Fallow deer samples not successfully amplified with certain markers.

OheS	C32	C105	C276	WY68	WY82	SD08	Capcap37
D_02	D_02	D_02	D_02	D_02	D_02	D_02	D_22
			D_03				D_23
							D_25
							D_26
							D_27

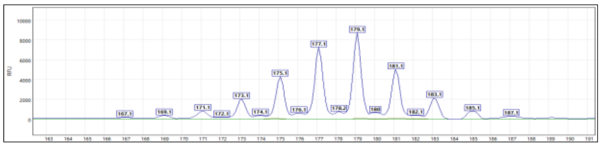

SBTD07_D33

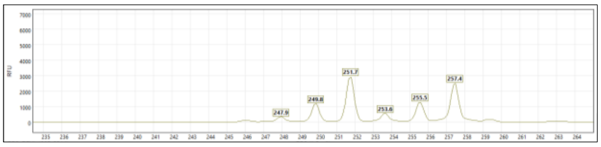

WY62_D05

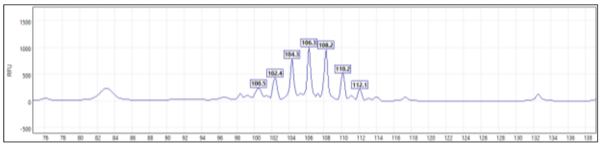

Capcap2_D10

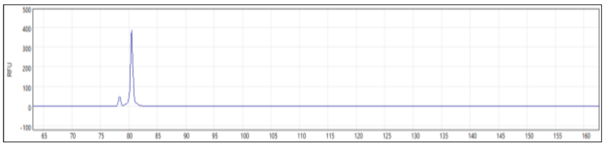

Capcap2_D23

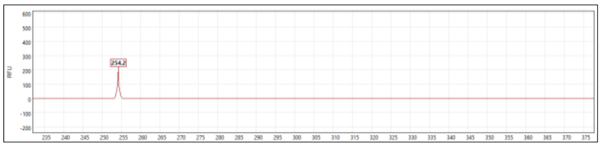

WY82_D22_run#1

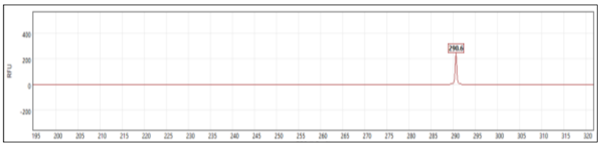

WY82_D22_run#2

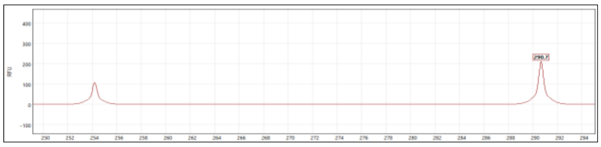

WY82_D30

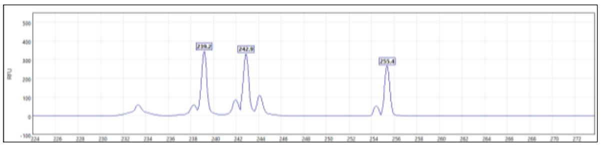

ApoV43_D03 v2

Figure S2. Markers excluded from further analysis based on the electrophoretic pattern.

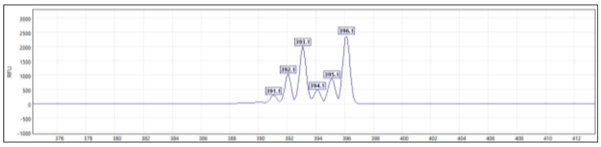

ApoV49_D48

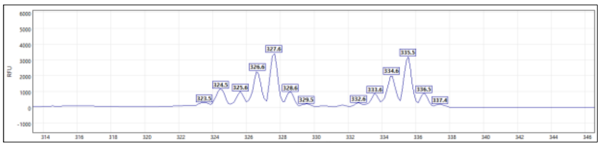

ApoV75-pigtail_D28

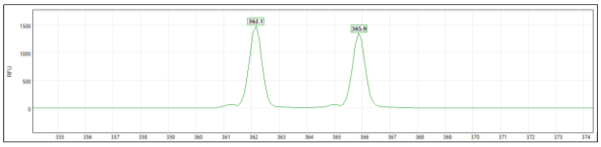

C276_D27

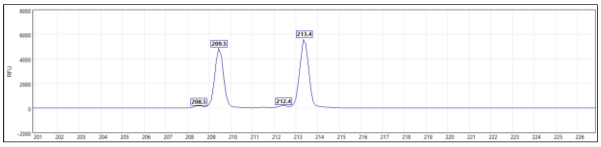

T268_D30

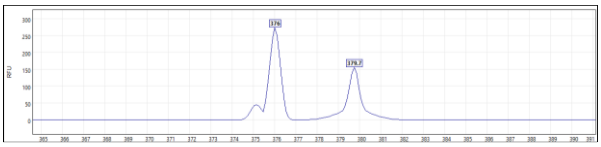

C276_D10

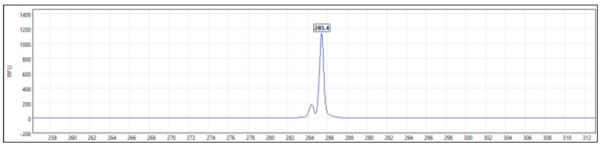

C32_D48

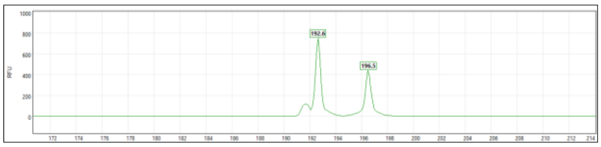

Mgou20_D40

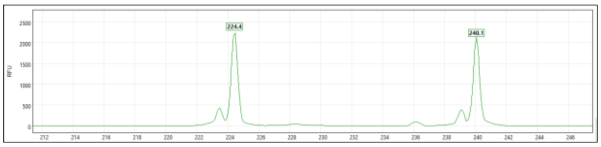

T268_D33

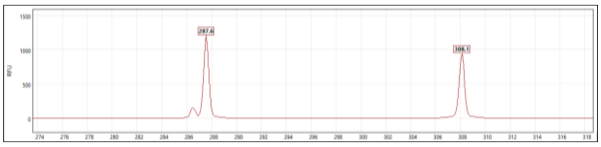

T107_D08

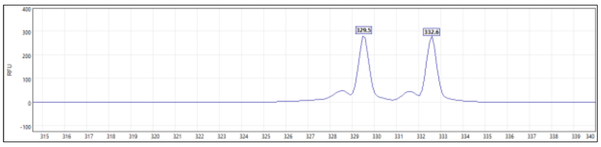

ApoV47_D40

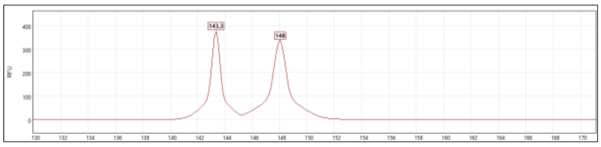

ApoV146_D10

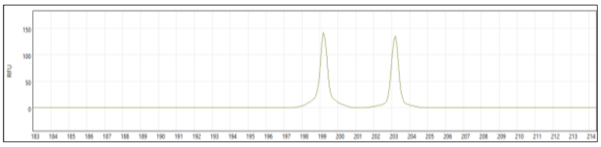

Capcap29_D25

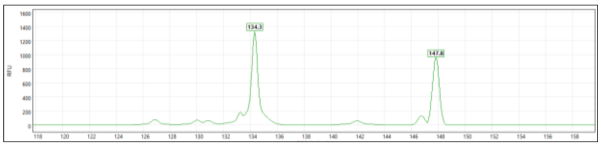

T156_D06

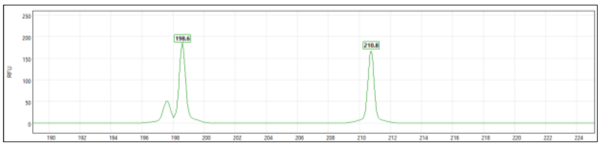

OheF_D25

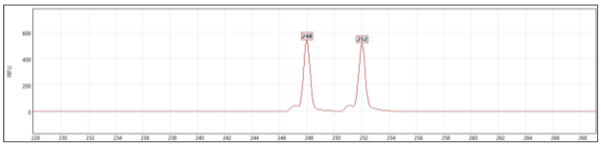

OheQ_D23

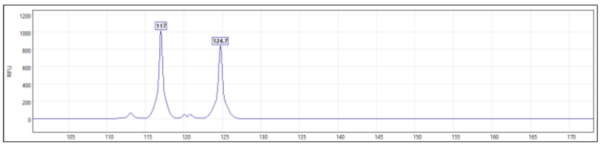

C229_D03

Figure S3. 14 polymorphic markers included in further statistical analysis based on their electrophoretic pattern.

Table S11. Genotypes and statistical analysis of 20 fallow deer samples based on 14 polymorphic microsatellite markers. Pop: population, n: number of alleles, PIC: polymorphic information content, H(obs): observed heterozygosity, H(exp): expected heterozygosity, N: number of samples.

Sample	Pop	OheF		OheQ		C229		T156		Capcap29		T107		ApoV47		T268		C276		ApoV49		Mgoua20		ApoV146		C32		ApoV75	
D_01	PIL	199	211	248	248	117	117	135	135	199	199	284	304	329	329	224	224	376	380	412	412	193	193	144	144	289	289	321	329
D_02	PIL	199	199	248	248	117	125	135	135	199	199	280	304	329	329	224	224	0	0	412	412	193	197	144	144	0	0	321	329
D_03	PIL	199	199	248	248	117	125	135	135	199	199	284	288	329	329	224	224	0	0	412	412	197	197	144	148	289	289	321	329
D_05	PIL	199	199	248	248	117	125	135	135	199	199	284	308	329	329	224	240	376	376	412	412	197	197	144	148	289	289	329	329
D_06	PIL	199	211	248	248	117	125	135	147	199	203	284	308	329	329	224	224	376	376	412	412	193	193	144	144	289	289	321	321
D_08	PIL	199	199	248	252	117	117	135	135	199	199	288	308	329	329	224	224	376	376	409	412	193	197	144	148	289	289	321	321
D_10	PIL	199	199	248	248	117	117	135	135	199	199	284	304	329	329	224	228	376	380	409	412	193	197	144	148	289	289	321	321
D_21	VER	199	199	248	248	117	117	135	135	199	199	284	284	329	329	224	224	376	380	412	412	193	197	144	148	289	289	321	321
D_22	VER	199	199	248	248	117	117	135	135	199	199	284	284	329	329	224	224	376	380	412	412	193	197	144	148	289	289	321	329
D_23	VER	199	199	248	252	117	117	135	135	199	199	284	284	329	329	224	224	376	380	412	412	193	193	148	148	289	289	321	329
D_25	ISA	199	211	248	248	117	117	135	135	199	203	284	308	329	333	224	224	376	376	412	412	193	193	144	148	289	289	321	321
D_26	ISA	199	199	248	248	117	117	135	147	199	199	284	304	329	329	224	224	376	380	409	409	197	197	148	148	289	289	321	321
D_27	ISA	199	199	248	248	117	117	135	135	199	199	280	308	329	333	224	224	376	380	412	412	193	197	144	148	289	289	321	321
D_28	ISA	199	199	248	248	117	125	135	135	199	203	280	304	329	333	224	240	376	380	412	412	193	197	144	148	289	289	321	329
D_30	ISA	199	199	248	248	117	125	135	135	199	199	284	304	329	333	224	228	376	376	409	412	193	193	144	144	289	289	321	321
D_33	ISA	199	199	248	248	117	125	135	135	199	203	284	308	329	329	224	240	376	380	409	412	193	193	144	144	289	289	321	329
D_36	PIL	199	199	248	248	117	117	135	135	199	199	284	304	329	333	224	224	376	376	412	412	193	193	148	148	289	289	321	321
D_40	SZE	199	199	248	248	125	125	135	135	199	199	284	304	329	333	224	224	380	380	412	412	193	197	144	144	285	285	321	321
D_48	LAJ	199	199	248	248	117	117	135	135	199	199	304	304	333	333	224	224	376	376	409	412	193	193	144	144	289	289	321	321
D_49	LAJ	199	199	248	252	125	125	147	147	199	199	288	304	329	329	224	224	376	376	409	412	193	197	144	144	285	285	321	321
allele		199		248		117		135		199		280		329		224		376		409		193		144		285		321	
n; allele freq.		37	0,925	37	0,925	29	0,725	36	0,900	36	0,900	3	0,075	32	0,800	35	0,875	25	0,694	8	0,200	25	0,625	25	0,625	4	0,105	31	0,775
allele		211		252		125		147		203		284		333		228		380		412		197		148		289		329	
n; allele freq.		3	0,075	3	0,075	11	0,275	4	0,100	4	0,100	17	0,425	8	0,200	2	0,050	11	0,306	32	0,800	15	0,375	15	0,375	34	0,895	9	0,225
												288				240													
												3	0,075			3	0,075												
												304																	
												11	0,275																
												308																	
												6	0,150																
																													**Mean**
n		40		40		40		40		40		40		40		40		36		40		40		40		38		40	
PIC		0,129		0,129		0,319		0,164		0,164		0,665		0,269		0,214		0,334		0,269		0,359		0,359		0,171		0,288	**0,274**
H(obs)		0,150		0,150		0,350		0,100		0,200		0,800		0,300		0,250		0,500		0,300		0,450		0,450		0,000		0,350	**0,311**
H(Exp)		0,142		0,142		0,409		0,185		0,185		0,728		0,328		0,232		0,437		0,328		0,481		0,481		0,193		0,358	**0,331**
N		20		20		20		20		20		20		20		20		18		20		20		20		19		20	

Table S12. Summary of Chi-Square Tests for Hardy-Weinberg Equilibrium on our markers. DF: degree of freedom, ChiSq: chi-square value, Prob: probability, Signif: significance (ns=not significant, * P<0.05, ** P<0.01, *** P<0.001)

Locus	DF	ChiSq	Prob	Signif
**OheF**	1	0.131	0.717	ns
**OheQ**	1	0.131	0.717	ns
**C229**	1	0.299	0.585	ns
**T156**	1	3.951	0.047	*
**Capcap29**	1	0.247	0.619	ns
**T107**	10	8.417	0.588	ns
**ApoV47**	1	0.078	0.780	ns
**T268**	3	0.408	0.939	ns
**C276**	1	0.571	0.450	ns
**ApoV49**	1	0.078	0.780	ns
**Mgoua20**	1	0.032	0.858	ns
**ApoV146**	1	0.032	0.858	ns
**C32**	1	19.000	0.000	***
**ApoV75**	1	0.000	0.987	ns
